# From Photon Beam to Accelerated Particle Beam: Antimetastasis Effect of Combining Radiotherapy With Immunotherapy

**DOI:** 10.3389/fpubh.2022.847119

**Published:** 2022-03-29

**Authors:** Liqiu Ma

**Affiliations:** ^1^Department of Nuclear Physics, China Institute of Atomic Energy, Beijing, China; ^2^National Innovation Center of Radiation Application, Beijing, China

**Keywords:** accelerated particle beam, photon beam, metastasis, radiotherapy, immunotherapy, abscopal effect

## Abstract

Cancer is one of the major diseases that seriously threaten the human health. Radiotherapy is a common treatment for cancer. It is noninvasive and retains the functions of the organ where the tumor is located. Radiotherapy includes photon beam radiotherapy, which uses X-rays or gamma rays, and particle beam radiotherapy, using beams of protons and heavy ions. Compared with photon beam radiotherapy, particle beam radiotherapy has excellent dose distribution, which enables it to kill the primary tumor cells more effectively and simultaneously minimize the radiation-induced damage to normal tissues and organs surrounding the tumor. Despite the excellent therapeutic effect of particle beam radiotherapy on the irradiated tumors, it is not an effective treatment for metastatic cancers. Therefore, developing novel and effective treatment strategies for cancer is urgently needed to save patients with distant cancer metastasis. Immunotherapy enhances the body's own immune system to fight cancer by activating the immune cells, and consequently, to achieve the systemic anticancer effects, and it is considered to be an adjuvant therapy that can enhance the efficacy of particle beam radiotherapy. This review highlights the research progress of the antimetastasis effect and the mechanism of the photon beam or particle beam radiotherapy combined with immunotherapy and predicts the development prospects of this research area.

## Introduction

Cancer is one of the leading causes of death worldwide (1). About 10 million people died of cancer in 2020 (2). Approximately 90% of cancer-related deaths are caused by distant metastasis (3). Early-stage cancer can be cured by surgery, chemotherapy, and radiotherapy. However, once the cancer has progressed to the advanced stage, the cancer cells spread to other organs; hence, even if the primary tumor can be effectively treated, distant metastasis becomes the key problem affecting the patient's survival and quality of life. Therefore, the development of novel and effective treatment strategies for cancer is urgently needed to treat patients with distant metastasis.

Considerable progress has been achieved in cancer treatment research in recent years (4). With the development of nuclear science and technology, the emerging concept of “precise treatment of tumors by nuclear technology” has gradually been recognized, and the most representative treatment concept is particle beam radiotherapy based on protons and heavy ions (5). Particle beam radiotherapy differs from traditional radiotherapy in terms of its physical and biological characteristics. It possesses excellent depth-dose distribution, which accurately and efficiently induces cancer cell death with limited effect on the surrounding healthy tissue (5), thereby avoiding or reducing the occurrence of complications during the treatment. However, as cancer progresses, the cancer cells are able to enhance their metastatic capacity and escape from immune surveillance, eventually leading to the formation of metastatic cancers in distal organs (6, 7). Particle beam radiotherapy has a excellent therapeutic effect on the irradiated tumors, but this treatment is ineffective for micrometastatic cancer, which is not diagnosed by CT and PET (8–11). Therefore, scientific studies, which are designed to suppress or even cure metastatic cancer, are imperative for enhancing the efficacy of particle beam radiotherapy.

The probability of the induction of the abscopal effect during radiotherapy in clinical practice is negligible. Usually, it can bring survival benefit to patients who have already developed distant metastatic cancer. The abscopal effect was first proposed by Mole in 1953 (12). This phenomenon refers to the regression of distant tumor lesions that have not received radiotherapy following local radiation exposure to the tumor. Presently, the primary manner of deepening one's understanding of abscopal effect is via a small number of published clinical case reports and preclinical studies about the induction mechanism of this effect. Most of the early case reports focused on the abscopal effect that was induced only by local photon radiotherapy (13–17). Some recent case reports have confirmed that both proton beam and heavy ion beam radiotherapy are also able to induce the abscopal effect (18, 19). However, studies on the induction mechanism of the abscopal effect remain limited. For instance, the study conducted by Camphausen et al. using the Trp53-deficient mice suggested that, at the molecular level, the induction of the abscopal effect was dependent on the tumor suppressor gene p53 (20). Overall, the abscopal effect is a very rare phenomenon in clinical practice, and its induction mechanism remains unclear.

Immunotherapy is considered as an auxiliary mean to enhance the efficacy of radiotherapy. The most representative types of immunotherapy include the “dendritic cell (DC)-based vaccine therapy”, advocated by Nobel Prize winner Ralph M. Steinman (21), and the “immune checkpoint inhibitor therapy” (22, 23) developed based on the “immunotherapy targets PD-1 and CTLA-4” discovered by Nobel Prize winners Tasuku Honjo and James P. Allison. Immunotherapy is dependent on the patient's immune system to fight cancer cells; thus, it is a systemic cancer treatment, and it is expected to bring hope to patients with cancer metastasis.

This review discusses the research progress of the antimetastasis effect and its mechanism of photon beam or particle beam radiotherapy combined with immunotherapy, and predicts the future developmental trend.

## Photon Beam Radiotherapy Combined With Immunotherapy

Once immunotherapy is combined with other cancer treatments, its remarkable synergy improves therapeutic effects significantly (24–26). The combination of radiotherapy and immunotherapy has become a research focus in tumor treatment, including photon beam radiotherapy combined with DCs or immune checkpoint inhibitors. DCs are a type of antigen-presenting cells that possess the function of antigen internalization and presentation, wherein they present antigens to T cells, which triggers the systemic antitumor immune response in the body (27). The antitumor and antimetastasis effects have been shown in preclinical studies regarding photon beam radiotherapy combined with DCs (28, 29). On the other hand, attention has also been paid to photon beam radiotherapy combined with immune checkpoint inhibitors (30–33). Immune checkpoint molecules, like PD-1 and CTLA-4, can negatively regulate the antitumor immune responses in the body by inducing a decrease in the activation level of killer T cells, which enables cancer cells to escape the attack of immune cells. The treatment with immune checkpoint inhibitors blocks the signal transduction of the immune checkpoint molecules on the surface of the T cell membrane, releasing the anticancer “brake” of the immune system, which is expected to produce effective and lasting activation of antitumor immunity in the body. The *New England Journal of Medicine* reported a successful case of combining conventional radiotherapy with CTLA-4 immune checkpoint inhibitor for the treatment of pleural-based paraspinal metastatic melanoma, wherein the regression of hilar lymphadenopathy and splenic metastatic lesions was observed (30). This report also found an increase in the proportion of immune-activating CD4^+^ICOS^high^ lymphocytes in the blood but a decrease in the proportion of immunosuppressive myeloid-derived suppressor cells following radiotherapy. A similar case showed that the combination therapy, which was used to treat liver metastatic lung adenocarcinoma, led to the regression of liver, lung, and sacrum metastatic lesions and caused significant changes in the proportion of immune cells in the blood and carcinoembryonic antigen concentration after treatment (31). In addition to a few clinical cases, there have also been some basic studies of radiotherapy combined with immune checkpoints. For instance, when photon beam radiotherapy was used in combination with the dual immune checkpoint inhibitors (anti-CTLA-4 and anti-PD-L1), the effects of these two immune checkpoint inhibitors did not cause redundancy, but instead they complemented each other through different immune activation mechanisms, thereby inhibiting the growth of the unirradiated distant metastatic tumor and improving survival (32). Another study revealed that the photon beam radiotherapy combined with the dual immune checkpoint inhibitors (anti-CTLA-4 and anti-PD-L1) had a better antimetastasis efficacy than radiotherapy combined with either CTLA-4 or PD-L1 immune checkpoint inhibitor alone (33). In summary, the combination of photon beam radiotherapy and immunotherapy can potentially inhibit cancer metastasis.

Although radiotherapy combined with immune checkpoint inhibitors brings excellent antitumor and antimetastasis effects, the side effects caused by immune checkpoint inhibitors cannot be underestimated. The CTLA-4 and PD-1/PD-L1 inhibitors lead to side effects such as rash, diarrhea, hepatotoxicity, and endocrine disorders and an overall incidence of adverse drug events of above 50% (34, 35). Apart from the abovementioned side effects of immune checkpoint inhibitors, when they are used in combination with radiotherapy, some other side effects have also been reported. The journal of *JAMA Oncology* reported that in the treatment of brain metastasis, which comes from primary melanoma, non-small-cell lung cancer, and renal cell carcinoma, symptomatic radiation necrosis of the brain occurred in approximately 20% of the patients who received radiotherapy combined with immune checkpoint inhibitors, whereas, this occurred in only 6.8% of the patients who only received radiotherapy (36). Another report indicated that a history of radiation pneumonitis prior to PD-1 immune checkpoint inhibitor treatment can increase the risk of interstitial lung disease (37). Therefore, to ensure both the safe use and antimetastasis effect of the combination therapy in more patients, safety studies for the combination therapy need to be conducted from a novel perspective.

## Particle Beam Radiotherapy Combined With Immunotherapy

With the development of nuclear science and technology, preclinical studies regarding particle beam radiotherapy combined with immunotherapy for metastasis suppression have also been conducted. Particularly, much attention has been given to the preclinical study of particle beam radiotherapy combined with DC-based immunotherapy, performed by the research team of the National Institute of Radiological Sciences (Japan). In 2010, this team reported that combining particle beam radiotherapy with α-galactosylceramide (α-GalCer) pulsed DC-based immunotherapy could effectively inhibit the formation of metastatic cancer (38). Later on, this team reported that in particle beam radiotherapy combined with DC-based immunotherapy, metastatic cancer suppression could be achieved by intravenous injection of the immature DC amplified *in vitro*, without the need of loading the DC with α-GalCer. This finding revealed that the killing effect of natural killer T (NKT) cells might not be involved in the primary mechanism of the antimetastasis effect of the combination therapy (39). This study also demonstrated that DC-based immunotherapy combined with particle beam radiotherapy was more effective in suppressing cancer metastasis than when combined with photon beam therapy at equivalent biological effect doses of photon beam or particle beam irradiation. In a recent study conducted by this team, the universality of the abovementioned combination therapy was verified using mice with different genetic backgrounds and their syngeneic types of cancer (40). The results demonstrated that the combination therapy effectively inhibited lung metastasis in Th1-dominant mice but not in Th2-dominant mice. Further analysis of the activation level of DCs suggested that the Th balance-related host genetic background rather than the tumor affected the antimetastasis effect of particle beam radiotherapy combined with DC-based immunotherapy. These results have provided a basis for the clinical screening of patients who are suitable for receiving combination therapy, ensuring that combination therapy is performed on the appropriate patients to achieve better treatment outcomes and its usage is avoided for patients who are not suitable for it.

Moreover, preclinical studies of particle beam radiotherapy combined with immune checkpoint inhibitors have been conducted. For instance, a preclinical study performed by the team of Osaka University (Japan) reported that particle beam radiotherapy combined with dual immune checkpoint inhibitors (anti-CTLA-4 and anti-PD-L1) effectively suppressed both the proliferation of primary tumors and unirradiated distant tumors (41). According to the study jointly conducted by GSI Helmholtzzentrum für Schwerionenforschung GmbH (Germany) and National Institute of Radiological Sciences (Japan), particle beam combined with dual immune checkpoint inhibitors (anti-CTLA-4 and anti-PD-1) induced a higher antimetastasis effect compared with photon beam at the equivalent dose of same primary tumor control level (42). The above two studies have revealed that particle beam radiotherapy combined with dual immune checkpoint inhibitors exhibit potential sensitized antitumor and antimetastasis effects, and may be more effective than the combination of photon beam and dual immune checkpoint inhibitors.

The abovementioned studies have suggested that particle beam radiotherapy combined with immunotherapy is a novel and effective treatment strategy for patients with cancer metastasis, but its universality and safety data are limited and need to be further investigated in preclinical studies and clinical settings in the future.

## Induction of the Abscopal Effect by Radiotherapy Combined With Immunotherapy

Immunotherapy is a new method of cancer treatment that has been developing in recent years. Local radiotherapy combined with immunotherapy has been performed to induce the abscopal effect in an increasing number of clinical cases. For instance, several clinical cases have reported that the abscopal effect is induced by combining radiotherapy with immune checkpoint inhibitors (30, 31). Furthermore, a clinical trial showed that local radiotherapy combined with the subcutaneous injection of granulocyte-macrophage colony-stimulating factor (GM-CSF) demonstrated a higher probability of inducing the abscopal effect on non-small cell lung cancer, breast cancer, and thymic cancer. Moreover, the cases with induced abscopal effect showed a longer overall survival in most patients (43).

Most of the preclinical studies have focused on the induction of the abscopal effect by combining radiotherapy with immunotherapy. For instance, photon beam radiation exposure combined with FMS-like tyrosine kinase 3 ligand (Flt3-L) on mice bearing 67NR breast cancer induces the abscopal effect (44). Another study showed that radiotherapy combined with tumor-associated antigen specific vaccine induced the regression of unirradiated distant tumors and pulmonary metastases (45). A series studies reported that 6 MeV electron beam therapy combined with chemokine CCL3 variant (eMIP) could also significantly induce the regression of unirradiated distant tumors, and the subsequent study showed that following the implementation of this combination therapy, eMIP combines with the radiation-induced damage-associated molecular patterns *in vivo* to activate CD8^+^ and CD4^+^ T cells and natural killer (NK) cells, which are then involved in inducing the abscopal effect (46–48). Recently, multiple studies by using dual tumors-bearing mouse model regarding immune checkpoint inhibitors have been reported, and their findings suggested that radiotherapy combined with immune checkpoint inhibitors can also induce the abscopal effect. For instance, melanoma B16-F10 tumor-bearing mice were treated with photon beam radiotherapy combined with anti-CTLA-4 antibody immunotherapy. After 3 weeks of radiotherapy, 17% of the mice showed complete response (CR) (32). In addition, photon beam combined with dual immune checkpoint inhibitors (anti-CTLA-4 and anti-PD-L1) applied to mice bearing LM8 osteosarcoma showed more than 40% of the mice with CR (33). Furthermore, compared with photon beam, particle beam combined with dual immune checkpoint inhibitors (anti-CTLA-4 and anti-PD-1/PD-L1) immunotherapy showed an increased proportion of mice with CR (41) and an enhanced inhibition of lung metastasis (42). Some studies on the underlying mechanism for antitumor immunity indicated that particle beam radiation has the ability to induce immunogenic cell death in cancer cells, releasing “eat me” and “danger” signals, such as calreticulin (CALR) and high mobility group box 1 (HMGB1) (49, 50), respectively, thereby promoting the activation of DCs, which ultimately activates antitumor immunity (39). Therefore, immunotherapy is expected to enhance particle beam radiation-induced antitumor immunity, thereby increasing the induction efficiency of the abscopal effect.

**Figure 1:**
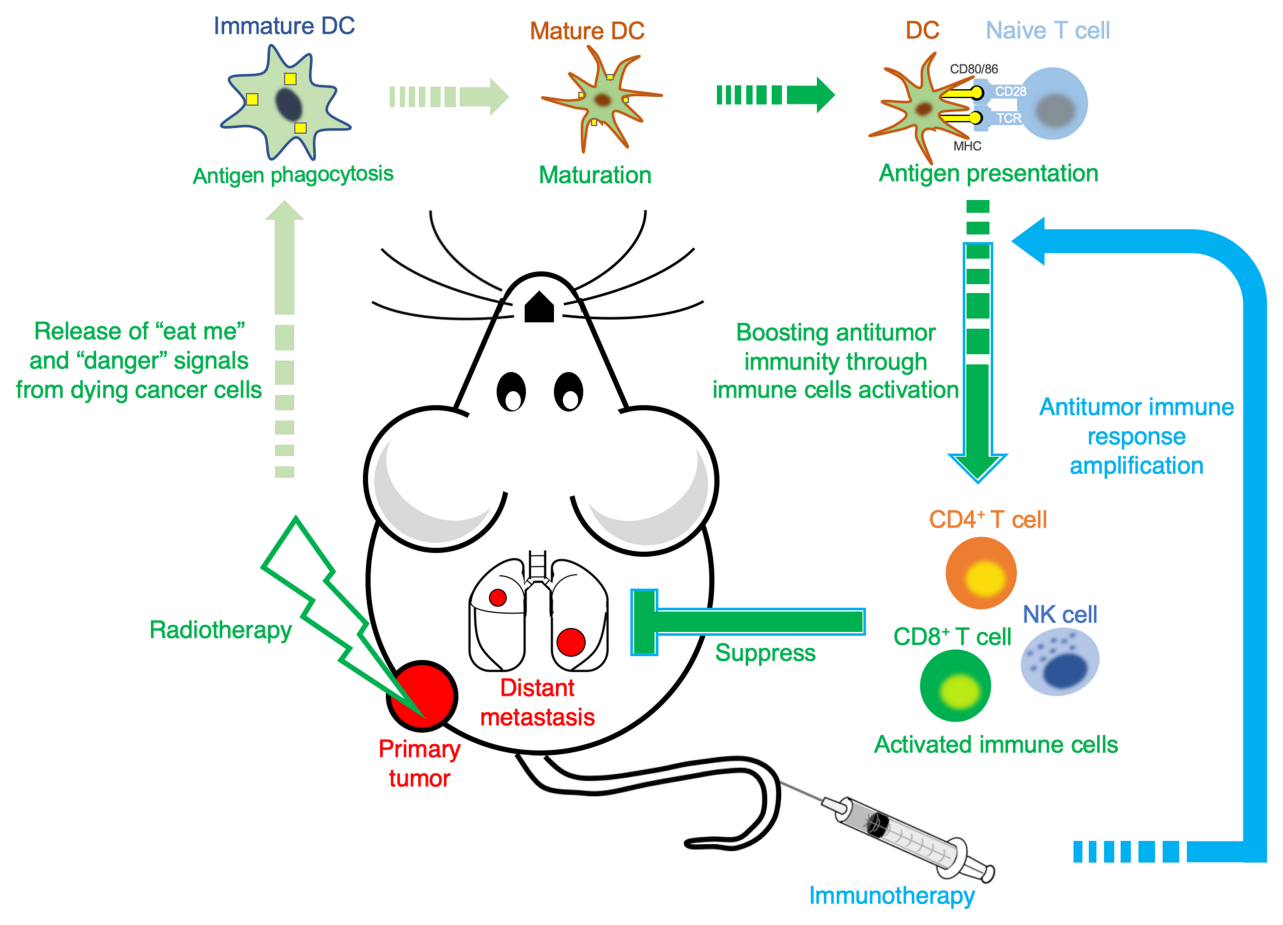
A possible mechanism of antitumor immunity by radiotherapy combined with immunotherapy. Following local tumor irradiation, “eat me” signal (cell surface calreticulin) and “danger” signals (such as high mobility group box 1 and adenosine triphosphate) are released from the dying cells and introduced directly by the dendritic cells (DCs). The DCs gradually mature after phagocytizing antigens, and then activate and direct them to present the tumor antigens to T cells, which are active and direct immune cells, to locate out and suppress both local tumor and distant metastasis. Immunotherapy further amplifies this antitumor immune response.

In summary, the radiation-induced activation of antitumor immunity may be a key factor in inducing the abscopal effect and immunotherapy further amplifies this immune response and enhances the systemic anticancer effect (Figure 1). The stable induction and deep-level mechanism of the abscopal effect can be studied by using the combination of radiotherapy and immunotherapy. Through these studies, it is expected that the abscopal effect can be turned from a rare phenomenon into a stable and effective tool for antimetastasis therapy in the near future.

## Discussion

Optimizing the radiation parameters of radiotherapy combined with immunotherapy is expected to engender better treatment outcomes. A recent study of radiotherapy combined with anti-CTLA-4 antibody immunotherapy showed that the relatively large total radiation dose that can almost eradicate the primary tumor not only significantly improved the treatment outcomes of the irradiated primary tumor but also inhibited the proliferation of the unirradiated distant tumor (51). Another study showed that combining multifractionated radiotherapy with CTLA-4 immune checkpoint inhibitor induced the abscopal effect better than combining single-fractionated radiotherapy with CTLA-4 immune checkpoint inhibitor (52). Additionally, optimizing the physical parameters of radiotherapy has the potential of reducing the risk of side effects caused by the combination therapies. A mice study conducted by the University of Pennsylvania found that the radiation dose in proton therapy was an important factor affecting hematologic toxicity (53). Vozenin et al. have proven that ultra-high dose rate (FLASH) radiotherapy could significantly reduce acute skin reactions in an animal study using cats (54). Whole abdominal proton FLASH radiotherapy was performed on mice by Diffenderfer et al., and it significantly inhibited intestinal fibrosis compared with standard proton radiotherapy (55). Although there are no current reports on FLASH radiotherapy combined with immunotherapy, the advantages of local tumor control and lower toxic side effects of FLASH radiotherapy indicate that this combination therapy may play a more important role in the future of cancer treatment. Therefore, optimizing the physical parameters of radiotherapy, such as radiation dose, dose rate, and dose fractionation, in combination therapy is associated with both antitumor and antimetastasis effects, as well as the toxic side effects on healthy tissues and organs. The best therapeutic effect of combination therapy is essentially the maximization of the anticancer effect and the minimization of the toxic side effects.

**Figure 2:**
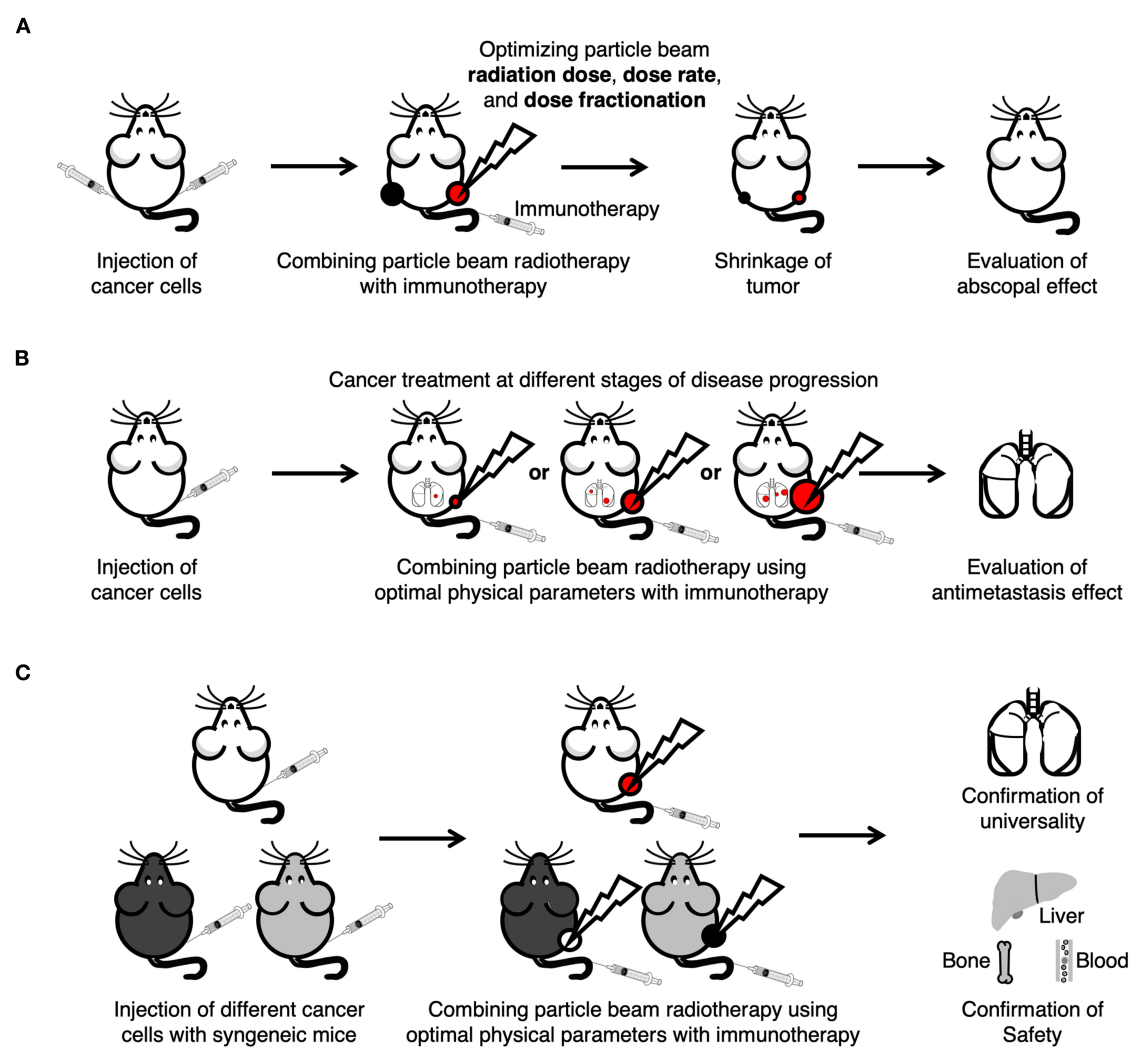
Future preclinical antimetastasis studies of particle beam radiotherapy combined with immunotherapy. **(A)** Enhancement of the abscopal effect by optimizing the particle beam physical parameters, such as radiation dose, dose rate and dose fractionation. **(B)** Evaluation of antimetastasis effects that correspond to different primary tumor disease progression levels after combining particle beam radiotherapy using optimal physical parameters with immunotherapy. **(C)** Confirmation of universality and safety of combination therapy by tumor-bearing syngeneic mice models.

In both photon beam radiotherapy and particle beam radiotherapy, a significant shrinkage or even regression of unirradiated distant metastatic tumor has been observed in some individual patients after local irradiation of the tumor, and this phenomenon is called the abscopal effect (18, 19, 30). An increasing number of evidence has suggested that the abscopal effect is mediated by the immune system after radiation (30, 44, 56). However, abscopal effect is extremely rare, and current studies mainly focus on the very few clinical case reports and induction mechanism of the effect. To date, although irradiation of cancer cells by particle beam can activate the antitumor immune response (39), no evidence has been found that particle beam radiotherapy alone can stably induce the abscopal effect. Particle beam radiotherapy combined with immunotherapy opens novel perspectives regarding the stable induction of the abscopal effect. For future preclinical studies of particle beam radiotherapy combined with immunotherapy, the following prospects are proposed (Figure 2). First, the efficiency of inducing the abscopal effect should be improved by optimizing the physical parameters of radiation in the combination therapy. Second, the antimetastasis effect should be evaluated after combining particle beam radiotherapy using optimal physical parameters with immunotherapy. Finally, the universality and safety of the combination therapy should be researched to provide a theoretical basis and data support for conducting future clinical trials. Once the combination therapy that can stably induce the abscopal effect has been found, cancer patients with distant metastasis may be eventually cured by receiving particle beam radiation to kill the primary tumor, and then inducing the abscopal effect with the appropriate methods to eliminate the metastatic cancer.

## Author Contributions

LM drafted the manuscript, contributed to the literature search and analysis, and reviewed and approved the final manuscript.

## Funding

This research was supported by the CIAE President's Strategic Grant (No. YZ212404000801).

## Conflict of Interest

The author declares that the research was conducted in the absence of any commercial or financial relationships that could be construed as a potential conflict of interest.

## Publisher's Note

All claims expressed in this article are solely those of the authors and do not necessarily represent those of their affiliated organizations, or those of the publisher, the editors and the reviewers. Any product that may be evaluated in this article, or claim that may be made by its manufacturer, is not guaranteed or endorsed by the publisher.
